# Mechanical Stress Induces VEGF Expression and RPE Disruption in Mouse Eyes

**DOI:** 10.3390/biology15090664

**Published:** 2026-04-22

**Authors:** Akira Minamoto, Ji-Ae Ko, Kota Haruyama, Atsushige Ashimori, Kazuhiro Kimura, Yoshiaki Kiuchi, Hirokazu Sakaguchi

**Affiliations:** 1 Department of Ophthalmology and Visual Science, Graduate School of Biomedical and Health Sciences, Hiroshima University, 1-2-3 Kasumi, Minami-ku, Hiroshima 734-8551, Japan; mi7moto@hiroshima-u.ac.jp (A.M.); kota073000@gmail.com (K.H.); ykiuchi@hiroshima-u.ac.jp (Y.K.); sakaguh@hiroshima-u.ac.jp (H.S.); 2 Department of Ophthalmology, Yamaguchi University, Yamaguchi 755-8505, Japan; a-ashi@yamaguchi-u.ac.jp (A.A.); k.kimura@yamaguchi-u.ac.jp (K.K.)

**Keywords:** retinal pigment epithelium, mechanical stress, VEGF, neovascular AMD, mouse model

## Abstract

Mechanical stress has been implicated in retinal pigment epithelium (RPE) dysfunction and angiogenic signaling in retinal disorders; however, its direct in vivo effects on the RPE–choroid complex remain incompletely understood. Here, we established a mouse model of localized mechanical stress by subconjunctival implantation of glass beads (0.8–1.2 mm in diameter) in eight-week-old *C57BL/6J* mice to induce transscleral stretching of the RPE. Ocular tissues were analyzed two days after implantation using histological, immunohistochemical, and molecular approaches, and inflammatory mediators were quantified by multiplex cytokine assays. Mechanical stress induced focal serous retinal detachment, elongation of photoreceptor outer segments, and disruption of the RPE tight junction protein ZO-1. VEGF expression in the RPE–choroid complex was significantly upregulated and accompanied by increased levels of inflammatory mediators, including MCP-1. Intravitreal administration of anti-VEGF agents effectively suppressed stress-induced VEGF expression. These findings indicate that mechanical stress is sufficient to induce structural disruption and angiogenic signaling in the RPE in vivo, providing a useful experimental platform for investigating stress-related retinal responses and therapeutic modulation of VEGF signaling.

## Simple Summary

Mechanical forces are increasingly recognized as important regulators of cellular behavior; however, their role in retinal diseases remains incompletely understood. In neovascular age-related macular degeneration, physical stress on the retinal pigment epithelium (RPE), caused by structural changes such as drusen accumulation, has been suggested to contribute to disease progression. In this study, we developed a mouse model that applies localized mechanical stress to the RPE by inserting small glass beads beneath the conjunctiva. This mechanical stimulation induced reproducible structural alterations in the retina and RPE, including disruption of cell junctions and increased expression of vascular endothelial growth factor (VEGF) in the RPE–choroid complex. Importantly, VEGF upregulation was effectively suppressed by intravitreal administration of clinically used anti-VEGF agents. This experimental system provides a simple and reproducible in vivo platform for investigating how mechanical stress influences RPE function and angiogenic signaling and may be useful for evaluating therapeutic responses under mechanically stressed conditions.

## 1. Introduction

The maintenance of retinal structure and function depends on the integrity of specialized epithelial cells, including the retinal pigment epithelium (RPE), which plays a central role in supporting photoreceptors and regulating the choroidal environment. Dysregulation of RPE function is implicated in several retinal disorders, most notably age-related macular degeneration (AMD), a leading cause of vision loss worldwide in individuals over 50 years of age [1,2]. AMD is characterized by progressive retinal degeneration affecting the macula, which is responsible for central, high-acuity vision [3]. Clinically, AMD is classified into atrophic (dry) and neovascular (wet) forms, the latter involving pathological angiogenesis beneath the retina [4,5,6]. Neovascular AMD is associated with choroidal neovascularization (CNV), leading to subretinal and intraretinal fluid accumulation, hemorrhage, and damage to macular structures essential for vision [5]. The pathogenesis of neovascular AMD is complex and multifactorial, involving aging, genetic factors, and extracellular deposits (drusen) located between Bruch’s membrane and the RPE [5,7,8]. Among these factors, RPE dysfunction is considered a key contributor to disease progression, although the underlying mechanisms remain incompletely understood.

Under physiological conditions, the RPE maintains retinal homeostasis in part by regulating vascular development in the choroid [9,10]. However, when RPE function is compromised, angiogenic factors such as vascular endothelial growth factor (VEGF) can become dysregulated, promoting the development of CNV [11,12,13,14]. Drusen contribute to RPE dysfunction by inducing oxidative stress, inflammation, and VEGF expression [15,16]. In addition to these biochemical factors, mechanical stress imposed on the RPE by drusen-associated structural alterations has emerged as a potential contributor to disease pathogenesis. In vitro studies have shown that mechanical stress applied to RPE cells induces the expression of pro-angiogenic and pro-inflammatory mediators, including VEGF, angiopoietin-2 (Ang2), hypoxia-inducible factor-1α (HIF-1α), interleukin-6 (IL-6), and interleukin-8 (IL-8) [17,18,19,20]. These findings suggest that mechanical stress may represent an underexplored mechanism contributing to angiogenic and inflammatory responses in the RPE.

Several animal models have been developed to study neovascular AMD, including laser-induced CNV models [21,22] and genetically modified mouse models [23]. However, these models do not specifically address the direct effects of mechanical stress on the RPE. To address this limitation, our group recently developed a mouse model in which localized mechanical stress is applied to the RPE by subconjunctival insertion of glass beads. Using this model, we previously demonstrated upregulation of VEGF and Ang2 mRNA in the RPE, supporting the concept that mechanical stress can modulate angiogenic signaling [19].

Building on these findings, the present study aimed to further characterize this model by analyzing structural changes in the retina and RPE, as well as alterations in angiogenic and inflammatory factors. This study focuses on mechanistic observations rather than disease modeling. By elucidating the effects of mechanical stress on the RPE in vivo, we seek to provide a useful experimental platform for investigating stress-related retinal responses and therapeutic modulation of angiogenic signaling.

## 2. Materials and Methods

### 2.1. Materials

Mouse monoclonal antibodies against rhodopsin were purchased from Millipore (Billerica, MA, USA). Rabbit polyclonal antibodies against ZO-1 were obtained from Thermo Fisher Scientific (Waltham, MA, USA). Antibodies against VEGF were sourced from GeneTex (Irvine, CA, USA), and those against MCP-1 were acquired from Cell Signaling Technology (Danvers, MA, USA). Goat polyclonal antibodies against Iba1 were purchased from Abcam (Cambridge, UK), and mouse monoclonal antibodies against α-tubulin were obtained from Sigma-Aldrich (St. Louis, MO, USA). Horseradish peroxidase–conjugated secondary antibodies were obtained from Promega (Madison, WI, USA). Alexa Fluor 488–conjugated secondary antibodies and 4′,6-diamidino-2-phenylindole (DAPI) were purchased from Molecular Probes (Carlsbad, CA, USA).

For intravitreal injections, a goat polyclonal antibody against mouse VEGF164 was obtained from R&D Systems (Minneapolis, MN, USA). Aflibercept (Eylea) was obtained from Bayer AG (Leverkusen, Germany), and faricimab (Vabysmo) was obtained from Chugai Pharmaceutical Co., Ltd. (Tokyo, Japan). Goat polyclonal IgG, used as a control, was also purchased from R&D Systems (Minneapolis, MN, USA). Intravitreal injections were performed with the following reagents. Mouse VEGF164 antibody and control IgG were injected at 5 µg/µL (1.5 µL per eye). Aflibercept (Eylea^®^) and faricimab (VABYSMO) were injected at 10 µg/µL (1.5 µL per eye).

### 2.2. Ethical Approval

All animal experiments were conducted in accordance with the guidelines of the Committee on Animal Experimentation of Hiroshima University and the Laboratory Animal Science Research Facility Committee of the Natural Science Center for Basic Research and Development, Hiroshima University (Approval No. A22-17), which functions as the Institutional Animal Care and Use Committee (IACUC). All procedures complied with institutional guidelines and were reported in accordance with the ARRIVE guidelines.

### 2.3. Animals and Tissue Preparation

Eight-week-old *C57BL/6J* mice were purchased from The Jackson Laboratory Japan, Inc. (Yokohama, Japan) and maintained under standard laboratory conditions at Hiroshima University.

General anesthesia was induced by intraperitoneal injection of a mixture of medetomidine (0.3 mg/kg), midazolam (4.0 mg/kg), and butorphanol (5.0 mg/kg). Topical anesthesia was achieved using 0.4% benoxinate hydrochloride eye drops prior to ocular manipulation.

Subconjunctival implantation of glass beads was performed to induce localized mechanical stress. Mice were analyzed at predefined time points of 2 and 3 days after bead implantation. Ocular tissues including the RPE–choroid complex were collected. Mice were euthanized by intraperitoneal overdose of thiopental sodium (300 mg/kg) in accordance with the AVMA Guidelines for the Euthanasia of Animals.

### 2.4. Histological Analysis (H&E Staining)

Ocular tissues were fixed in Neo-Fix solution (Merck, Darmstadt, Germany) at room temperature for 24 h. After fixation, tissues were dehydrated in graded ethanol, cleared in xylene, and embedded in paraffin. Sections (2 µm thick) were prepared using a microtome (Leica Microsystems, Wetzlar, Germany) and mounted onto slides. Hematoxylin and eosin staining was performed using standard protocols, and sections were examined under a light microscope.

### 2.5. Immunohistochemistry

Paraffin-embedded sections (2 µm) were deparaffinized, rehydrated, and washed with phosphate-buffered saline (PBS). Sections were blocked with 3% bovine serum albumin (BSA) and 0.1% Tween-20 in PBS for 1 h at room temperature and incubated with anti-rhodopsin antibody (1:200) for 1 h. After washing, sections were incubated with Alexa Fluor 488-conjugated secondary antibodies for 1 h. Nuclei were counterstained with DAPI.

### 2.6. RPE Flat-Mount Preparation and ZO-1 Junction Analysis

RPE flat mounts were prepared and immunostained for ZO-1 and Iba1. Tissues were blocked with 3% BSA and 0.1% Tween-20 in PBS for 1 h and incubated with primary antibodies against ZO-1 (1:500) and Iba1 (1:100). After washing, tissues were incubated with Alexa Fluor 488-conjugated secondary antibodies.

Fluorescence images were acquired at ×400 magnification under identical conditions. ZO-1-positive intercellular junctions were manually traced using the segmented line tool in ImageJ software (NIH). The total junction length within a defined region of interest (ROI) was measured after calibration and normalized to the ROI area, expressed as ZO-1 junction length density (µm/10^4^ µm^2^). Two images per eye were analyzed, and the mean value was used for statistical analysis. Image analysis was performed in a blinded manner with respect to experimental groups.

### 2.7. Photoreceptor Outer Segment Thickness Measurement

Photoreceptor outer segment thickness was quantified in rhodopsin-stained retinal sections using ImageJ software (NIH). In bead-implanted eyes, measurements were performed within a 100-µm-wide region where elongation of outer segments was observed, representing areas of prominent structural change. Ten measurements were taken at equal intervals, perpendicular to the RPE layer, and averaged to obtain a single value per eye.

In control eyes, measurements were obtained from a region located at the same distance from the optic nerve head using the same procedure. The mean value per eye was used for statistical comparison.

### 2.8. Immunoblot Analysis

Ocular tissues including the RPE–choroid–sclera complex were collected at the indicated time points. Tissues were lysed in radioimmunoprecipitation assay (RIPA) buffer and homogenized. After centrifugation, supernatants were subjected to SDS-PAGE and transferred onto nitrocellulose membranes.

Membranes were incubated with primary antibodies followed by HRP-conjugated secondary antibodies. Signals were detected using enhanced chemiluminescence reagents. Band intensities were quantified by densitometry and normalized to α-tubulin.

### 2.9. Multiplex Cytokine Assay

Aqueous humor samples were collected after bead implantation. Cytokine levels were analyzed using a multiplex assay (Proteome Profiler Mouse Cytokine Array Panel A, R&D Systems) according to the manufacturer’s instructions. Cytokines with signal levels below the detection limit were not included in quantitative analysis.

### 2.10. Quantitative PCR (qPCR) Analysis

Total RNA was extracted from ocular tissues including the RPE–choroid–sclera complex using an RNeasy kit (Qiagen, Venlo, The Netherlands). Reverse transcription was performed, and quantitative PCR was conducted using SYBR Green Master Mix on a QuantStudio 3 system (Thermo Fisher Scientific, Waltham, MA, USA). Gene expression levels were normalized to an internal control gene (*GAPDH*) and analyzed using the comparative Ct method.

### 2.11. Statistical Analysis

Data are presented as mean ± standard deviation (SD). Normality was assessed using the Shapiro–Wilk test. Comparisons between two groups were performed using paired two-tailed Student’s *t*-tests. Statistical analyses were conducted using JMP Student Edition version 18 (SAS Institute Inc., Cary, NC, USA). A *p*-value < 0.05 was considered statistically significant.

## 3. Results

### 3.1. Mechanical Stress Induces Structural Disruption in the RPE and Retina

**Figure 1:**
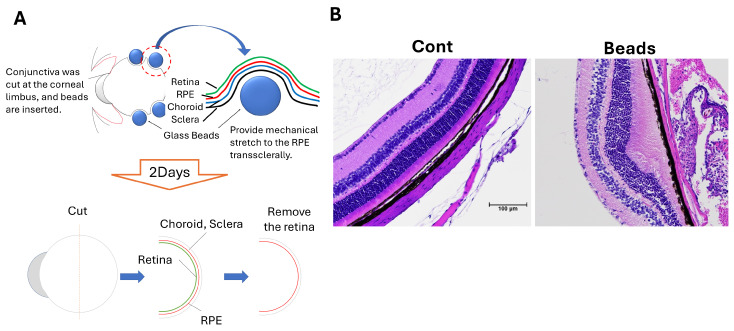
Mechanical stress alters retinal and RPE morphology following subconjunctival glass bead insertion. (**A**) Schematic illustration of subconjunctival glass bead insertion in the right eye of 8-week-old mice to apply localized mechanical stress to the retinal pigment epithelium (RPE). (**B**) Hematoxylin and eosin (H&E) staining of retinal sections showing elongation of photoreceptor outer segments in bead-implanted eyes compared with controls (scale bar: 100 μm).

To establish a model of localized mechanical stress, glass beads were inserted under the conjunctiva of the right eye in 8-week-old mice to induce stress on the RPE layer. Two days after implantation, ocular tissues including the RPE–choroid complex were collected for analysis (Figure 1A). Hematoxylin and eosin staining revealed elongation of photoreceptor outer segments in bead-implanted eyes compared with control eyes (Figure 1B).

**Figure 2:**
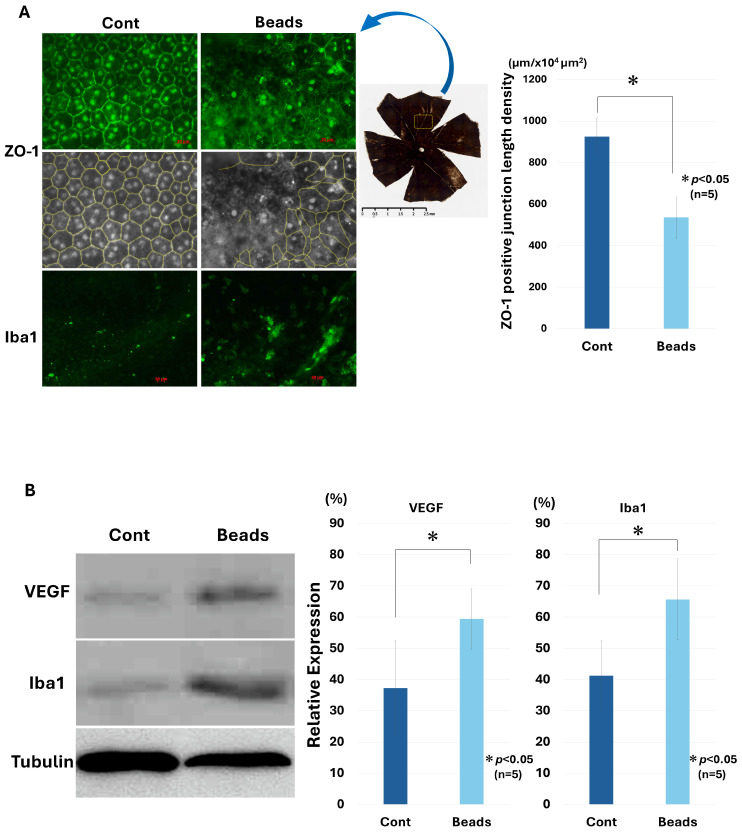
Disruption of ZO-1-positive junctions in RPE flat mounts following bead insertion. (**A**) Representative images of ZO-1 immunostaining in RPE flat mounts from control and bead-inserted eyes. In control eyes, ZO-1 staining outlines regular hexagonal RPE cell borders, whereas bead-inserted eyes exhibit disrupted and fragmented ZO-1-positive junctions. The middle panels show traced ZO-1 junctions used for quantification. Iba1 staining indicates increased inflammatory cell accumulation in bead-inserted eyes. The ZO-1-positive junctional length density (µm/10^4^ µm^2^) was significantly decreased in the bead-inserted eyes compared with the contralateral control eyes (924.64 ± 90.74 vs. 536.08 ± 101.23, n = 5, paired *t*-test, *p* < 0.05). (**B**) Western blot analysis showing increased expression of VEGF and Iba1 in bead-inserted eyes compared with control eyes. Tubulin was used as a loading control. Quantification of band intensities demonstrates significant increases in VEGF (33.68 ± 8.98% vs. 61.64 ± 12.84%) and Iba1 (38.78 ± 11.42% vs. 70.43 ± 12.15%) expression in bead-inserted eyes (n = 5, paired *t*-test, * *p* < 0.05 for both). Data are presented as mean ± SD.

Representative ZO-1-stained RPE flat mounts are shown in Figure 2A. In control eyes, ZO-1 staining delineated a regular hexagonal pattern of RPE cell borders. In contrast, bead-implanted eyes exhibited marked disruption of ZO-1-positive junctions, with irregular and fragmented cell borders. Quantitative analysis demonstrated a significant reduction in ZO-1 junction length density in bead-implanted eyes compared with controls (924.64 ± 90.74 µm/10^4^ µm^2^ vs. 536.08 ± 101.23 µm/10^4^ µm^2^, n = 5, paired *t*-test, *p* < 0.05). (Figure 2A). Western blot analysis further showed increased expression of VEGF and Iba1 in bead-implanted eyes. Quantitative analysis revealed that the relative expression levels (% of control) were significantly higher in bead-implanted eyes compared with contralateral control eyes for both VEGF (33.68 ± 8.98% vs. 61.64 ± 12.84%) and Iba1 (38.78 ± 11.42% vs. 70.43 ± 12.15%) (n = 5, paired *t*-test, *p* < 0.05 for both). (Figure 2B, Supplementary Figure S1). Consistently, increased Iba1 immunoreactivity was observed in the same regions (Figure 2A).

**Figure 3:**
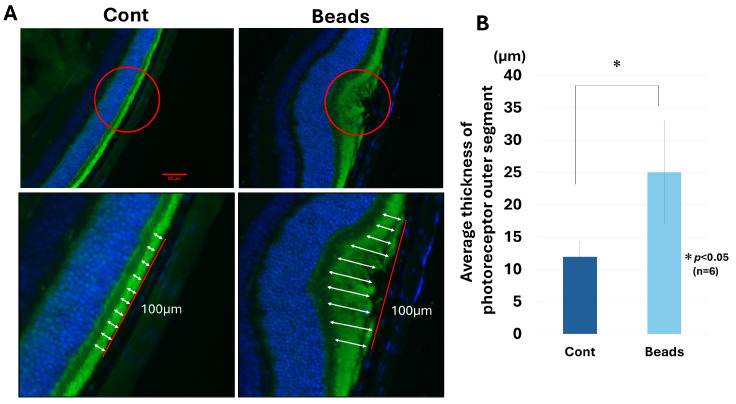
Elongation of photoreceptor outer segments in bead-inserted eyes. (**A**) Representative rhodopsin-immunostained retinal sections from control and bead-inserted eyes. The photoreceptor outer segments appear elongated in bead-inserted eyes (red circles). Lower panels show higher-magnification images. Outer segment thickness was measured within a 100-µm-wide retinal region (red line). Ten measurements were taken at equal intervals within this region and were performed perpendicular to the RPE layer (white arrows). Scale bar: 50 µm. (**B**) Quantitative analysis confirmed a significant increase in outer segment thickness (µm) in bead-implanted eyes compared with the contralateral control eyes (11.95 ± 2.39 µm vs. 25.04 ± 8.00 µm, n = 6, paired *t*-test, *p* < 0.05). Data are presented as mean ± SD.

Representative rhodopsin-immunostained retinal sections are shown in Figure 3A. Photoreceptor outer segments appeared elongated in bead-implanted eyes compared with controls. Quantitative analysis confirmed a significant increase in outer segment thickness (µm) in bead-implanted eyes compared with the contralateral control eyes (11.95 ± 2.39 µm vs. 25.04 ± 8.00 µm, n = 6, paired *t*-test, *p* < 0.05) (Figure 3B).

### 3.2. Time-Course Effects of Anti-VEGF Treatment in a Mechanical Stress Model

**Figure 4:**
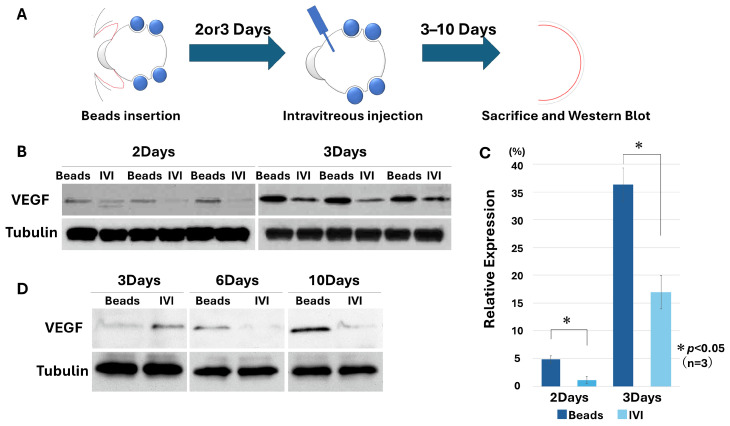
Time-course analysis of anti-VEGF effects in a bead-induced retinal stress model. (**A**) Schematic illustration of the experimental design for intravitreal anti-VEGF antibody injection following bead implantation. (**B**) Western blot analysis of VEGF expression at 2 and 3 days after bead implantation with or without anti-VEGF treatment. A greater reduction in VEGF expression was observed at day 3. (**C**) Quantification of VEGF protein levels by densitometric analysis of immunoblots. VEGF levels were normalized to α-tubulin. Relative VEGF expression (% of control) was significantly suppressed by intravitreal injection of an anti-VEGF antibody on both day 2 and day 3 (day 2: 4.81 ± 1.25% vs. 1.18 ± 0.67%; day 3: 36.30 ± 2.99% vs. 16.84 ± 4.83%) (n = 3, paired *t*-test, *p* < 0.05 for both). Data are presented as mean ± SD. Each data point represents the mean value per eye. (**D**) Extended time-course analysis of anti-VEGF effects at 3, 6, and 10 days after injection. The inhibitory effect on VEGF expression persisted for at least 6 days following treatment.

We next examined the time-course effects of anti-VEGF antibody injection at predefined time points. A reduction in VEGF expression was observed at both 2 and 3 days after bead implantation (day 2: 4.81 ± 1.25% vs. 1.18 ± 0.67%; day 3: 36.30 ± 2.99% vs. 16.84 ± 4.83%) (n = 3, paired *t*-test, *p* < 0.05 for both). Because the effect was more pronounced on day 3, subsequent analyses were focused on this time point (Figure 4B,C, Supplementary Figure S2). While similar trends were observed at both time points, the magnitude of the effect appeared greater at day 3. The effects of anti-VEGF treatment were further evaluated over time (3, 6, and 10 days post-injection) (Figure 4D, Supplementary Figure S2), demonstrating that VEGF suppression persisted for at least 6 days.

Validation of clinically used anti-VEGF agents and specificity of the observed effects.

**Figure 5:**
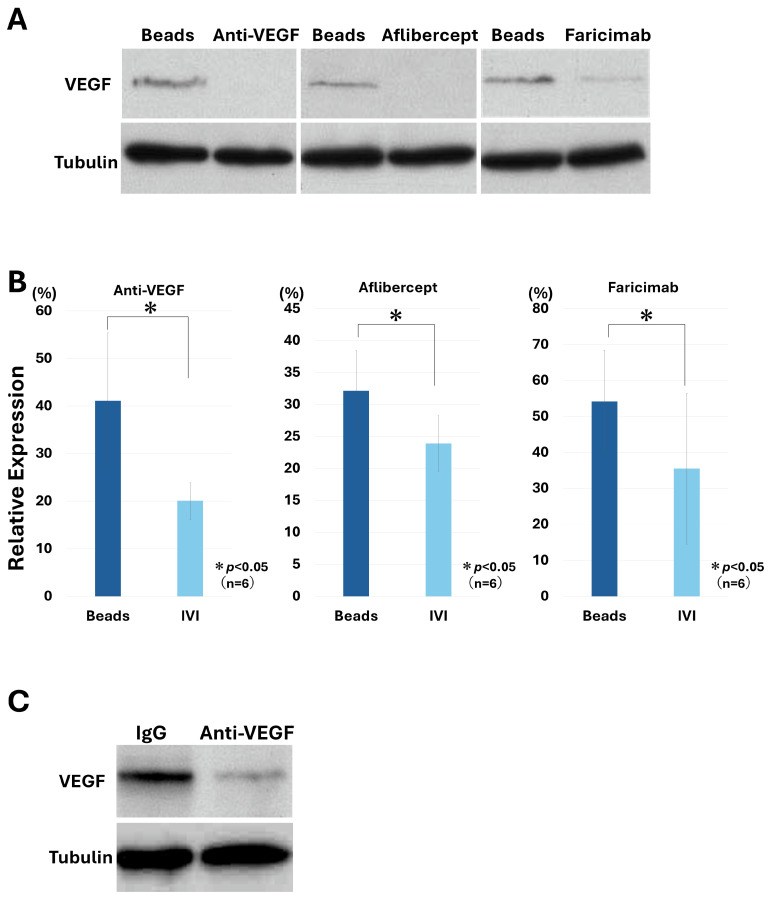
Clinically used anti-VEGF agents suppress VEGF expression in a bead-induced retinal stress model. (**A**) Western blot analysis of VEGF expression following intravitreal administration of clinically used anti-VEGF agents. All tested agents reduced VEGF expression. (**B**) Quantitative densitometric analysis of VEGF protein levels normalized to α-tubulin. The relative VEGF expression levels (% of control) were significantly reduced in the IVI groups compared with the beads groups for Anti-VEGF (41.11 ± 14.25% vs. 20.04 ± 3.88%), Aflibercept (32.12 ± 6.31% vs. 23.90 ± 4.31%), and Faricimab (54.17 ± 14.17% vs. 35.47 ± 20.98%) (n = 6 for each comparison, paired *t*-test, *p* < 0.05 for all), indicating a consistent suppressive effect across treatments. Data are presented as mean ± SD. (**C**) Western blot analysis following intravitreal injection of control IgG. VEGF expression was not reduced in the control group, confirming that the observed effects are specific to anti-VEGF treatment rather than non-specific effects of the injection procedure.

Given that anti-VEGF antibody treatment effectively suppressed VEGF expression in this model, we next examined the effects of clinically used anti-VEGF agents. Western blot analysis demonstrated that all tested agents reduced VEGF expression (Figure 5A, Supplementary Figure S3). Quantitative densitometric analysis confirmed a significant decrease in VEGF protein levels across all treatment groups (Figure 5B). The relative VEGF expression levels (% of control) were significantly reduced in the intravitreous injection (IVI) groups compared with the beads groups for Anti-VEGF (41.11 ± 14.25% vs. 20.04 ± 3.88%), Aflibercept (32.12 ± 6.31% vs. 23.90 ± 4.31%), and Faricimab (54.17 ± 14.17% vs. 35.47 ± 20.98%) (n = 6 for each comparison, paired *t*-test, *p* < 0.05 for all), indicating a consistent suppressive effect across treatments (Figure 5B).

To evaluate potential non-specific effects associated with the injection procedure, control IgG was administered intravitreally. VEGF expression was not reduced in the control IgG group (Figure 5C, Supplementary Figure S3), indicating that the observed effects were specifically attributable to anti-VEGF treatment rather than injection-related effects.

### 3.3. Inflammatory Response Associated with Mechanical Stress

**Figure 6:**
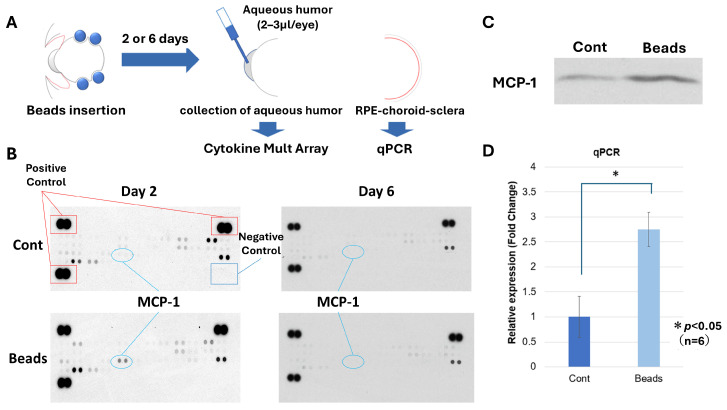
Mechanical stress induces MCP-1 upregulation in ocular tissues. (**A**) Schematic representation of the experimental design. (**B**) Multiplex cytokine analysis of aqueous humor samples showing increased MCP-1 levels in bead-implanted eyes compared with controls. MCP-1 levels decreased over time. (**C**) Western blot analysis confirming increased MCP-1 expression in bead-implanted eyes. (**D**) Quantitative PCR analysis of choroid–sclera tissue including the RPE demonstrating significant upregulation of MCP-1 mRNA at 2 days after bead insertion, normalized to *GAPDH*, of 2.75 ± 0.34 (n = 6, paired *t*-test, *p* < 0.05). Data are presented as mean ± SD.

To assess whether mechanical stress is associated with inflammatory responses, cytokine profiles were analyzed in aqueous humor samples. Multiplex cytokine analysis revealed a marked increase in MCP-1 levels in bead-implanted eyes compared with controls (Figure 6B).

This increase was further supported by Western blot analysis (Figure 6C, Supplementary Figure S4). In addition, quantitative PCR analysis using choroid–sclera tissue including the RPE demonstrated a significant upregulation of MCP-1 mRNA in bead-implanted eyes, with a relative expression level, normalized to *GAPDH*, of 2.75 ± 0.34 (n = 6, paired *t*-test, *p* < 0.05) (Figure 6D). MCP-1 levels in aqueous humor gradually decreased over time (Figure 6B). The mechanisms underlying this temporal decline remain unclear and warrant further investigation. These findings indicate that mechanical stress is associated with a transient inflammatory response in ocular tissues.

## 4. Discussion

In this study, we developed a mouse model in which localized mechanical stress applied to the retinal pigment epithelium (RPE) induces structural and molecular changes relevant to retinal pathology through subconjunctival glass bead insertion. This model reproducibly induced alterations in retinal and RPE morphology, including focal serous retinal detachment (SRD), elongation of photoreceptor outer segments, and disruption of the RPE tight junction protein ZO-1, accompanied by increased vascular endothelial growth factor (VEGF) expression (Figure 1 and Figure 2) [24,25,26,27]. These findings indicate that mechanical stress can directly affect RPE structure and function in vivo.

Our results suggest that mechanical stress is sufficient to disrupt RPE barrier integrity and trigger angiogenic and inflammatory responses. VEGF is a key mediator of choroidal neovascularization [11,12,13,14], and its upregulation in response to mechanical stress in this model supports the involvement of biomechanical factors in retinal pathology. The localized disruption of ZO-1 and induction of inflammatory markers, including Iba1, indicate that mechanical stress compromises RPE junctional stability and promotes focal inflammatory activation (Figure 2) [28,29,30]. Notably, these changes were spatially restricted to regions subjected to stress, consistent with the focal nature of retinal lesions.

With respect to therapeutic relevance, intravitreal anti-VEGF treatment produced a time-dependent suppression of VEGF expression in this model (Figure 4). Furthermore, clinically used anti-VEGF agents consistently reduced VEGF levels, whereas control IgG had no effect, confirming the specificity of the response (Figure 5). These findings support the utility of this model as an experimental platform for evaluating anti-angiogenic interventions under mechanically stressed conditions.

In addition to angiogenic signaling, mechanical stress was associated with a transient inflammatory response, characterized by increased monocyte chemoattractant protein-1 (MCP-1) expression in ocular tissues (Figure 6) [31,32]. The temporal decline in MCP-1 levels suggests an acute inflammatory response following mechanical stimulation. These findings support the concept that mechanical stress may act as an upstream trigger linking structural perturbation to inflammatory and angiogenic signaling in the RPE–choroid complex.

This study focuses on mechanistic observations rather than disease modeling. Although this model reproduces several features relevant to retinal pathology, it does not fully capture the chronic and multifactorial nature of neovascular AMD. Factors such as aging, long-term extracellular matrix remodeling, and genetic susceptibility were not addressed. In addition, analyses were limited to early time points and selected molecular markers, without long-term functional or imaging assessments. Nevertheless, the reproducible induction of VEGF and inflammatory responses highlights the utility of this model as a complementary approach to existing experimental systems. Also, HIF-1α is a well-established regulator of VEGF expression and has been reported to be responsive to various forms of cellular stress, including mechanical stimuli. Although HIF-1α was not examined in the present study, it may represent a potential upstream mediator of VEGF upregulation in this model. Further studies are warranted to investigate its involvement. [33,34].

In the present study, we focused on early molecular and structural responses of the RPE to localized mechanical perturbation. Functional assessments, such as electroretinography (ERG) or optical coherence tomography (OCT), were not included and would further strengthen the translational relevance of the model. Future studies incorporating these approaches will be important to determine the functional consequences of the observed changes.

## 5. Limitations

This study has several limitations. First, the analyses were restricted to early time points and primarily focused on structural and molecular endpoints, without long-term functional evaluation. Second, the magnitude and spatial distribution of mechanical stress applied to the RPE were not quantitatively defined, and the present model does not provide a direct measure of the mechanical strain experienced by the tissue. Therefore, the current analysis should be interpreted as a comparison between the presence and absence of mechanical perturbation rather than a quantitative dose–response relationship. Future studies incorporating imaging-based deformation analysis or computational modeling will be required to more precisely characterize the stress field. In addition, photoreceptor outer segment thickness was measured in regions showing prominent elongation, which may introduce selection bias and potentially overestimate the magnitude of structural changes. Future studies employing predefined sampling locations will be necessary to ensure unbiased and reproducible quantification. Third, the use of young *C57BL/6J* mice may limit generalizability to aged or diseased conditions. Despite these limitations, the model consistently induced VEGF upregulation and inflammatory responses, supporting its utility as a reproducible in vivo system for investigating stress-responsive pathways. In addition, a sham surgery control (e.g., conjunctival manipulation without bead insertion) was not included in this study. Therefore, it is difficult to fully distinguish the effects of mechanical stress from those of surgical manipulation or foreign-body-associated inflammation. This limitation is particularly relevant for the interpretation of inflammatory responses, such as MCP-1 upregulation and Iba1 expression. Future studies incorporating appropriate sham controls will be necessary to clarify the specific contribution of mechanical stress.

## 6. Conclusions

In conclusion, localized mechanical stress applied to the RPE induces reproducible structural disruption and angiogenic signaling in vivo. Rather than modeling the full spectrum of neovascular AMD, this system provides a focused experimental framework for investigating mechanical stress-driven RPE responses. This approach may contribute to a better understanding of how physical forces influence retinal biology and may support the development of therapeutic strategies targeting stress-related pathways.

## Data Availability

The original contributions presented in this study are included in the article/Supplementary Materials. Further inquiries can be directed to the corresponding author.

## Supplementary Materials

The following supporting information can be downloaded at: https://www.mdpi.com/article/10.3390/biology15090664/s1, Figure S1: Figure 2B Western blot, Figure S2: Figure 4B,D Western blot, Figure S3: Figure 5A,C Western blot, Figure S4: Figure 6C Western blot.
