# Supplementary figures and images for: Mechanical Stress Induces VEGF Expression and RPE Disruption in Mouse Eyes
Source: Biology (Basel). 2026 Apr 22;15(9):664. doi: 10.3390/biology15090664 (PMC13162610; doi:10.3390/biology15090664)

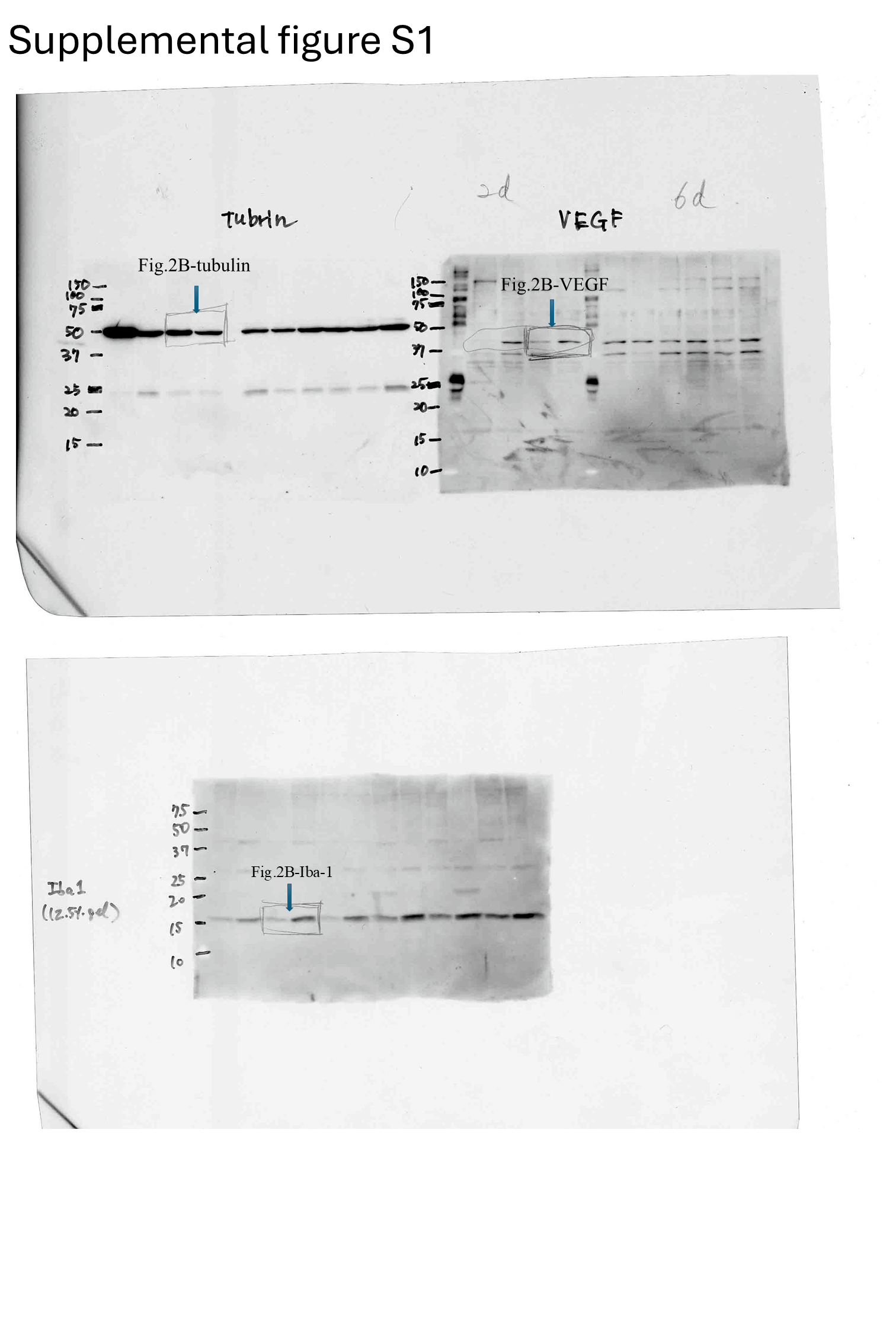

Supplement: Supplementary file 1 [file biology-15-00664-s001.zip › figure S1.png]

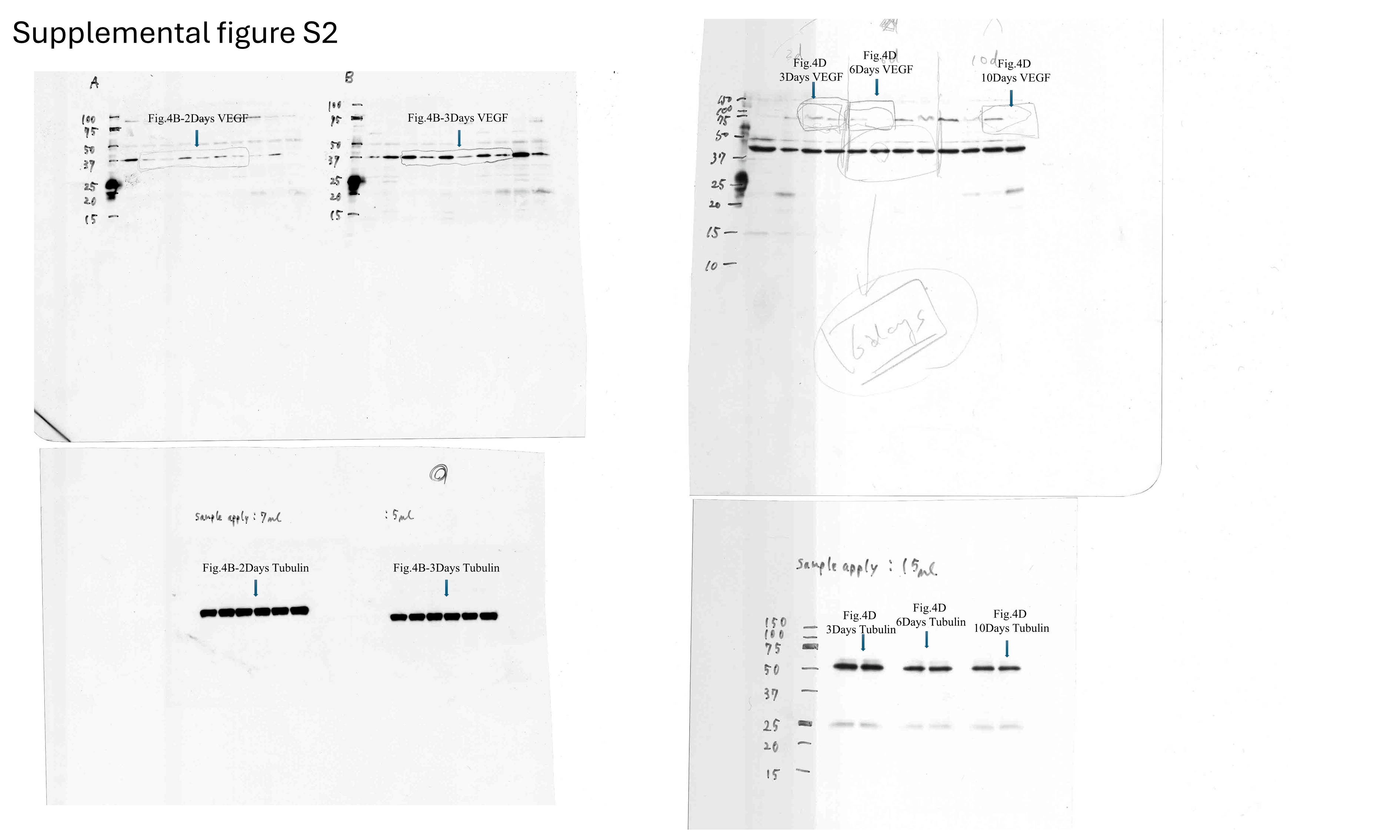

Supplement: Supplementary file 1 [file biology-15-00664-s001.zip › figure S2.png]

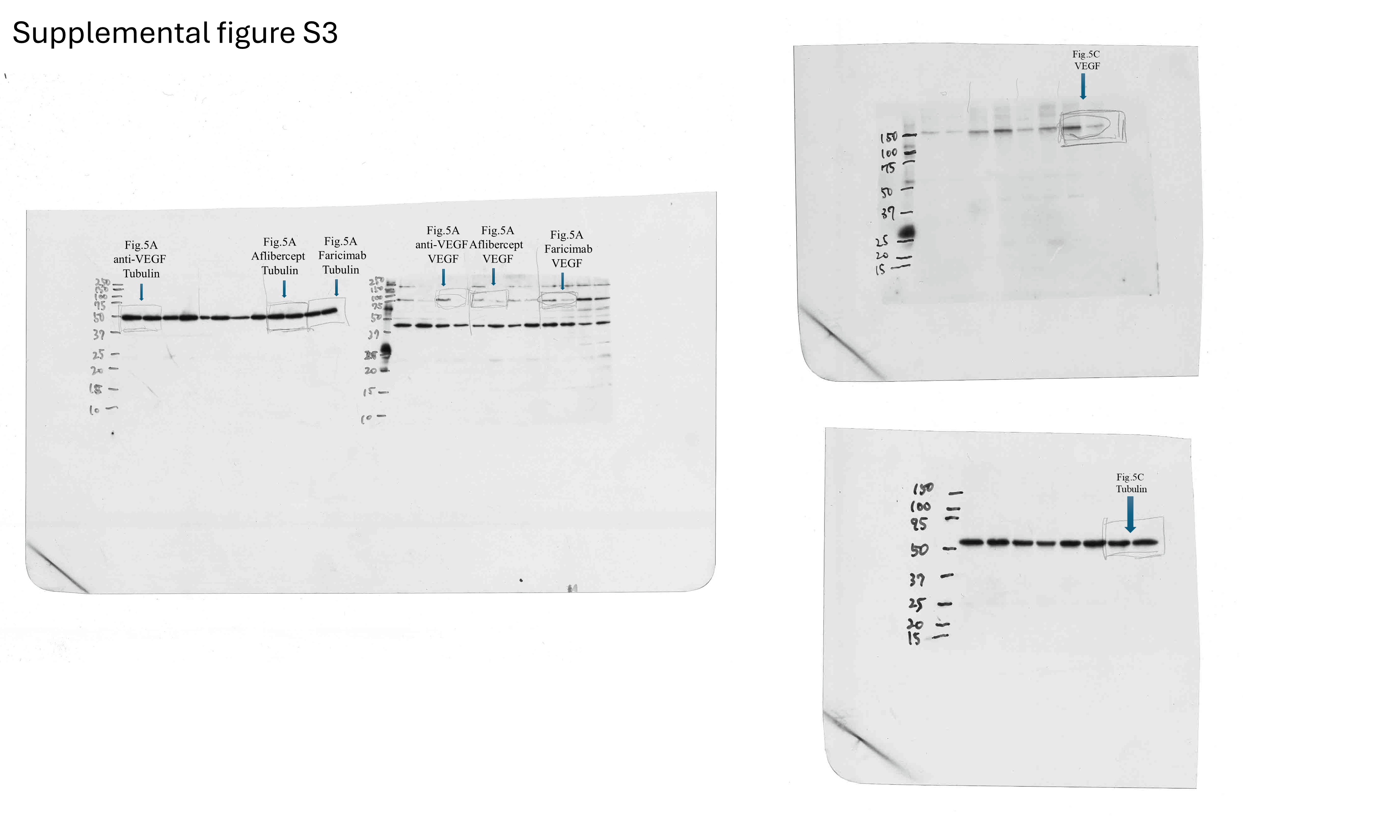

Supplement: Supplementary file 1 [file biology-15-00664-s001.zip › figure S3.png]

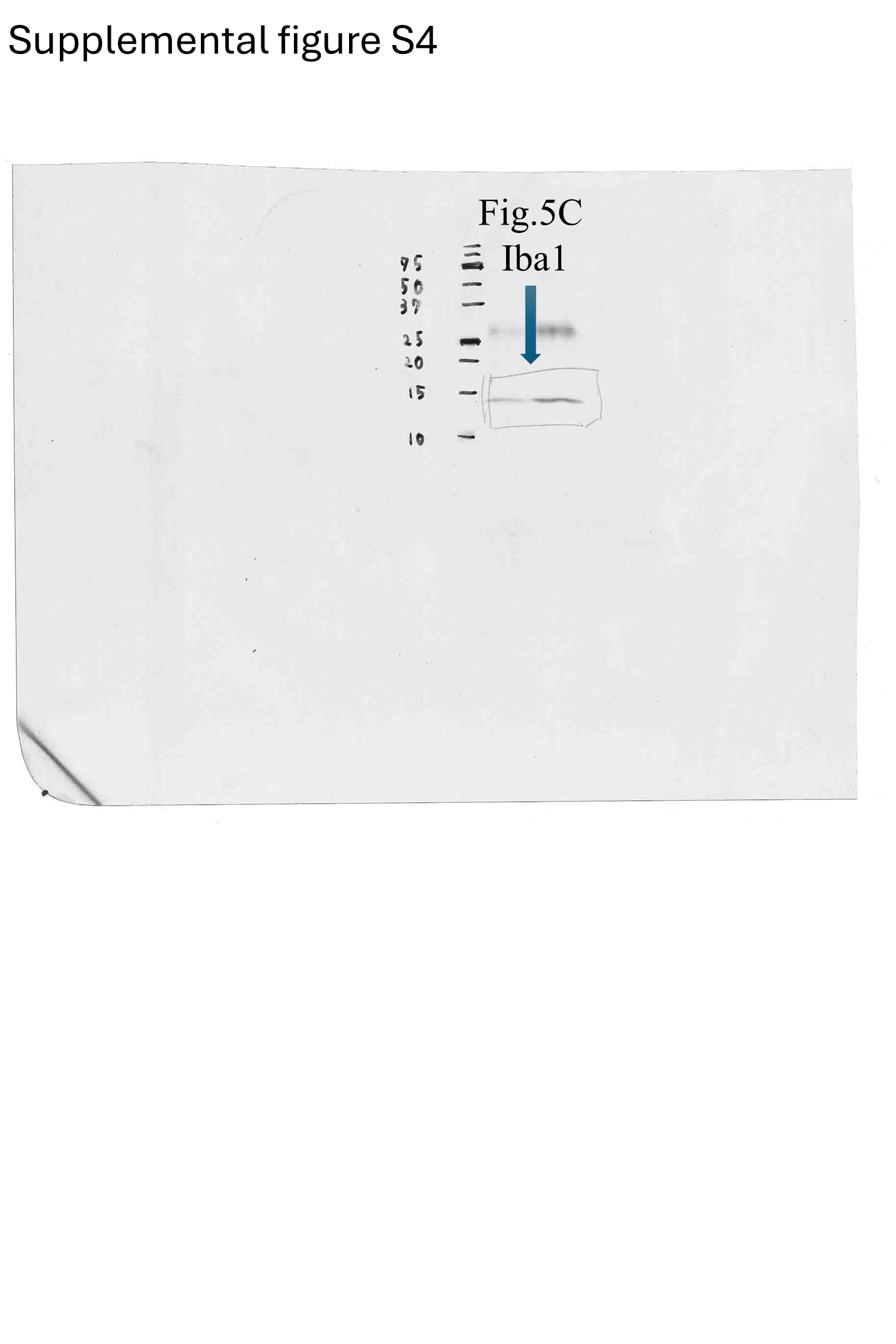

Supplement: Supplementary file 1 [file biology-15-00664-s001.zip › figure S4.png]
